# “Rhizosphere upheaval after tree cutting: Soil sugar flux and microbial behavior”

**DOI:** 10.1080/19420889.2022.2068110

**Published:** 2022-05-03

**Authors:** Enny Widyati, Ragil SB. Irianto, Adi Susilo

**Affiliations:** Forestry Research and Development Agency, Bogor, Indonesia

**Keywords:** Belowground sugar, coppice, cutting, functional groups, rhizosphere

## Abstract

Cutting trees removes all parts of their photosynthetic area, which affects rhizosphere assembly. However, information regarding the underground alteration process after tree cutting is insufficient. This study aimed to observe the fate of both root exudation and the rhizosphere microbial community following tree cutting. The study included 540 *Calliandra calothyrsus* Meissn. The experimental layout was a completely randomized block design with 3 blocks (cutting age) × 2 (cutting and not cutting) × 180 trees. Composite soil samples were collected from trees at 0–20 cm depth and stumps at 0, 2, 4, 8, and 12 weeks after cutting to observe the soil sugar content, pH, and functional group population. This study demonstrated that cutting reduced the flux of sugars below ground by 80% and caused rapid acidification (pH less than 5.0) of the soil. Total soil sugar depletion is presumed to be a mechanism by which *C. calothyrsus* survives and regrows after cutting. Sugar depletion affects significant shifts in the size and structure of the rhizosphere microbial community. Increasing soil acidity is another survival strategy to limit close competitor populations in the rhizosphere. This study confirms that *C. calothyrsus* is a proper species for developing in the coppice-harvesting-system (CHS) energy estate.

## Graphical abstract

**Figure:**
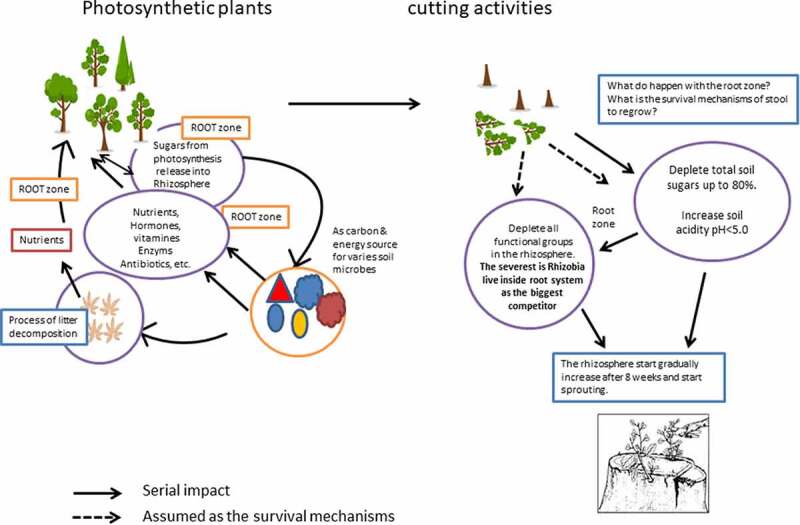

## Introduction

Plants are sessile organisms that interact intensively with soil microbes to accelerate growth and increase fitness [1,2]. Plants release diverse organic compounds from photosynthesis, estimated at 10–30% [3], collectively labeled as root exudates [4], which attract soil microbes and create an exclusive environment called the rhizosphere [5]. The rhizosphere is a critical zone because it is an arena for roots to access water and nutrients and interact with the physical and biotic components of the soil that influence plant growth [6].

In rhizosphere assemblage, plants initiate rhizosphere formation and control the structure and composition of root microbial communities. The function of root exudates is to appeal to and repel soil microbes such that the magnitude, structure, and composition of rhizosphere communities fit with the species, growth, and plant development stage [2,7]. Diverse types of soil microbes colonize the rhizosphere. Cellulose degraders decompose plentiful soil organic matter (SOM) in the rhizosphere. They are recognized as cellulolytic microbes because of their cellulase enzyme systems that degrade SOM [8]. Nitrogen fixers (NFs) are also essential microbial groups in the rhizosphere. Most N-fixing processes in terrestrial ecosystems are performed by symbiotic NF bacteria. The most popular is rhizobia, which builds symbioses with leguminous family plants, forming nodules in the roots. In the nodules, they differentiate into “bacteroid” forms for fixing atmospheric nitrogen and transforming it into ammonia. In return, plants obtain a “privileged” nitrogen source [9]. The legume tree rhizosphere is also found to be inhabited by free-living bacteria that play a critical role as plant growth promoters [10] and contribute to a significant amount of available nitrogen in the soil [11]. Nitrogen is commonly known as a macronutrient for the primary growth of plants, while for microbial communities this element is essential for protein synthesis. Phosphorus (P) is also a crucial macronutrient that limits plant productivity. Hence, the population of phosphate solubilizer (PS) microbes in the rhizosphere plays an essential role in providing P to the host plant [12].

Generally, cutting is supposed to be the most damaging interference to trees, as this action removes all plant sections functioning as photosynthetic areas. Photosynthesis is a crucial step in root exudate production. Specifically, 50% of root exudates in the rhizosphere are secreted as sugars [13], the principal carbon (C) source for microbes [14]. Therefore, tree cutting is assumed to also affect the interaction between plants and microbes in the rhizosphere. An earlier study found that cutting can successively reduce microbial biomass and indirectly influence the soil microbial community composition and structure [15–17]. Soil bacteria are highly responsive to soil nutritional changes [15,16,18], as these bacterial communities are mainly shaped by soil properties; hence, the dynamics of bacterial community structure and composition may present an obvious recovery trend after disturbances due to clear-cutting [19,20]. Earlier studies stated that forest harvest practice by clear-cutting removes more than the tree bole, significantly reducing total soil nitrogen and soil microbial biomass [21], restricting some sensitive taxa and changing community structure through niche selection [22], which can be viewed as a ‘reset’ of the community assembly or as an environmental filter [23]. The death of sensitive microbes after disturbance in forest soil may favor the growth of better-adapted microbes and cause a change in the microbial community and decomposition activity (Henesmaa et al., 24, 25]).

*Calliandra callotrysus* Meissn can regrow quickly after being completely cut. Therefore, in Indonesia, this species is recommended for short-rotation biomass development in a coppice harvesting system (CHS). This is a harvesting practice that involves regularly felling trees at the base, leaving 50–70 cm stumps to regrow. Specifically, CHS provides an earlier harvest, which gives return benefits to the farmers sooner. For instance, energy plantation forests in Indonesia are initially harvested at 12 to 24 months of age. After growing the copies, they can be cut every year.

However, information on the rhizosphere dynamics after cutting with the CHS and how the survival mechanisms of the stump regrow following this are still insufficient. This study examined the dynamics of the *C. calothyrsus* rhizosphere after cutting with CHS to answer the following questions: (i) how are the soil sugars, the most-secreted root exudates, fluctuating due to tree cutting; (ii) is there any alteration in the rhizosphere environment as a reaction of tree cutting; (iii) what are the dynamics of functional microbial populations in the rhizosphere after cutting until the coppice grows from the stump; and (iv) do the age of the plants at the time of felling affect the three phenomena previously asked? The results of this study provide the basis for potential beneficial forest plantation management, especially for the cultivation of short-rotation coppice species.

Since Indonesia is expected to be confronted with energy scarcity in the future due to fossil fuel resources dwindling, energy alternatives have to be provided. Tree biomass has the potential to be an environmentally friendly renewable source of energy. The cultivation of biomass energy is an initiative to give effort and support to reducing global warming through carbon assimilation.

## Material and methods

The study was conducted at the Majalengka company estate forest, in West Java Province, Indonesia (6°46ʹ44.618 “S, 108°17ʹ6.738 “E) from April to November 2019, at three different ages of *C. calothyrsus*, namely 12, 18, and 24 months old. The soil properties were classified as latosol and regosol, with an annual rainfall of 2,400–3,800 mm/y and eight rainy months.

The experimental design was a completely randomized block design with three replications, in which the blocking was based on tree age. A block consisted of 180 trees, which were then divided into two treatments.. cut (50–70 cm from the soil surface) and uncut (control), totaling 540 trees for this study. Observations of the rhizosphere were performed at 0, 1, 2, 4, 8, and 12 weeks after cutting.

Soil samples were collected from each stump at a depth of 0–20 cm using a shovel with a depth marker (0–30 cm). Sampling was performed only once per stump to ensure that variances were only from the tree-cutting treatment. In the first step, a hole of 40 × 40 × 40 cm was made, and on the rhizosphere side, we collected soil-free fine roots from the surface to a depth of 20 cm for approximately 1 kg. Moreover, 180 trees in each treatment block were randomly divided into six sampling times to obtain 30 composite samples per sampling time per block (consisting of 15 stumps and 15 uncut trees). The soil samples were transferred to the laboratory in cooler boxes to examine the following parameters..

1. *Total soil sugars (TSS) and soil acidity*

The TSS level was observed using high-performance liquid chromatography (HPLC) with the column separation method [5]. Soil acidity (pH of H_2_O) was measured using a pH meter by diluting the soil with aquadest to a ratio of 1:1 (w/v).

1. *The functional group of the soil microbe population*

The living soil microbial functional group population was indirectly calculated using the plate count method. This method was conducted through indirect criteria applied to evaluate active fractions of microbial biomass, including cultivability or staining with specific dyes [26].

The initial step involved the preparation of a soil solution in a series of dilutions. The 10 g of composite-sampled soil was diluted to 100 ml using 0.85% NaCl solution and was then set as the 10^−1^ dilution factor. One milliliter of 10^−1^ dilution was transferred into 9 ml of sterilized water using sterilized pipettes, resulting in a 10^−2^ dilution. In this same manner, a series of up to 10^−7^ dilutions were prepared under aseptic conditions. Isolation of CD and PS microbes was carried out in the 5^th^-7^th^ series, whereas isolation of free-living NF was conducted in the 4^th^-6^th^ series. These were performed using the following procedures..

### Isolation and calculation of cellulose degrading (CD) microbes

Soil samples with soil dilutions (1 ml) from the last three series were transferred to sterilized Petri dishes containing carboxymethyl cellulose (CMC) media stained with Congo Red [27]. The cultures were incubated for 3–7 d at 30°C. Cellulose degrader microbes were differentiated with clear zones around the colonies and counted. The CD microbes in this study will be considered as CMC digesters from here forward.

*Isolation and calculation of phosphate solubilizing (PS) microbes*

Soil dilutions (1 ml) from the last three series were transferred to sterilized Petri dishes containing Pikovskaya’s (PKV) agar media. The cultures were incubated for 3–7 d at room temperature. Phosphate solubilizing microbes were differentiated with clear zones around the colonies and counted [12].

### Isolation and calculation of free-living nitrogen fixing bacteria (NFB-FL)

In this study, the NFB-FL was represented by Azotobacter genera. Soil dilutions (1 ml) of the last three series were transferred to sterilized Petri dishes containing Azotobacter agar media. The cultures were incubated for 3–7 d at room temperature [9].

## Calculating effective nodules

Calculation of effective nodules, characterized by a white exterior and a pink to reddish interior color associated with leg-hemoglobin pigment, was conducted at three points on each tree sample within a radius of one meter from the stump. Each point was placed in a soil volume of [10 cm (length) × 10 cm (width) × 10 cm (depth)]. The edges of the sampling points were then dug up to 10-cm depth to limit contact with the surrounding soil and then sprayed with water to separate the roots from the soil. Root nodules with a white-pinkish color were calculated on an average from these three points.

## Data analysis

The percentage of alteration in either the root exudate concentration or soil microbial population was calculated using the following equation..

$$Alteration=\frac{\left(control-treatment\right)}{\left(control\right)}\times 100\%$$

Analysis of variance (ANOVA) for two-way factorial in a completely randomized block design was conducted to determine the main effects of two factors, cutting, and age, on root exudation and soil microbe populations using SPSS ver. 22 software. The test to determine the most influential factors was Duncan’s multiple range test at a 95% confidence level.

## Results

Table 1 demonstrates that cutting and sampling time significantly influenced TSS flux, while tree age did not. However, age, cutting, and sampling time had a significant effect on all functional groups of soil microbial communities in the rhizosphere, except for the number of effective nodules. Interactions among the treatments did not significantly affect any of the variables.

**Table 1.** Summary of ANOVA analysis (p-value) on the influence of tree-cutting on the total soil sugars and microbial communities in the rhizosphere*

Factors	Total soil sugar	Population of soil microbes			Number of nodules
		PS	CMC	BNF-FL	
Age (A)	0.753	0.000	0.000	0.000	0.000
Cutting (C)	0.000	0.000	0.000	0.000	0.000
Time of sampling (S)	0.026	0.000	0.000	0.000	0.080
(A*C)	0.372	0.714	0.467	0.689	0.211
(C*S)	0.991	0.364	0.295	0.236	0.969

* α = 0.05; df = 539.

### Soil sugars flux

**Figure 1.**
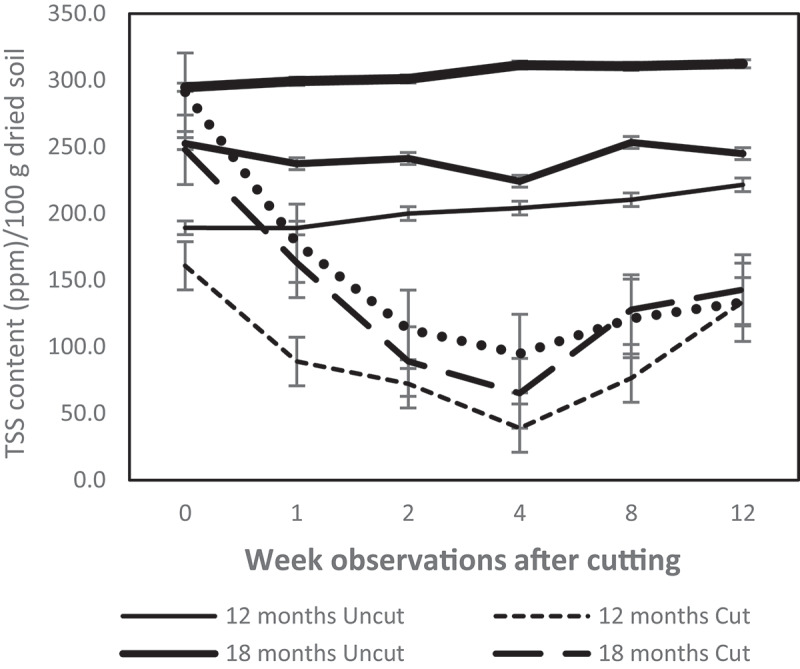
Dynamic of total soil sugar (TSS) in *C. calothyrsus* stump rhizosphere. The TSS contents reduce drastically a week after felling and continually deplete until the 4^th^ week. All cutting treatments show, the TSS start increasing in the 8^th^ week after felling. The TSS contents of the uncut group remain stable during the experiment. (Solid line: uncut trees; dotted line: cut trees).

Figure 1 demonstrates that cutting trees drastically decreased TSS release to the rhizosphere of *C. calothyrsus* at all ages of cutting. The TSS content in the rhizosphere was reduced a week after cutting and continued to decrease until the fourth week. In week four, TSS dramatically diminished by approximately 69–80%. Cutting 12-month old *C. calothyrsus* trees was the most susceptible. The TSS content of uncut trees remained stable until the twelfth week of observation (Figure 1).

### Rhizosphere acidity

**Figure 2.**
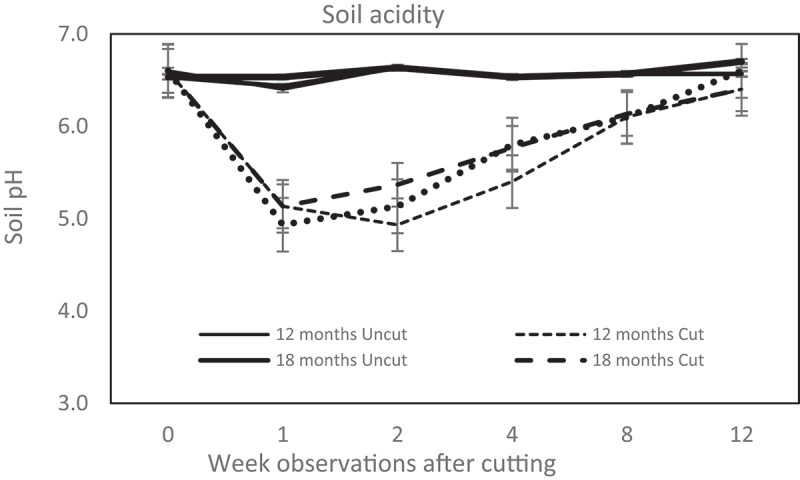
Dynamic of soil acidity in *C. calothyrsus* stump rhizosphere. All cut treatments show the soil pH immediately decreased a week after felling, except the group of 12 month age, the lowest pH happen at the 2^nd^ week after felling. The rhizosphere pH then gradually increased until at the 12^th^ week after cutting closer to the initial situation. There is no fluctuation in the soil acidity of the uncut treatments during the observations. (Solid line: uncut trees; dotted line: cut trees).

Figure 2 demonstrates that cutting trees increased rhizosphere acidity within a week, whereas the uncut group showed only a minor alteration. During the first week of observation, the pH of the rhizosphere stumps declined 1.5–1.7 units and was stable until the second week.

### Microbial dynamics

**Figure 3.**
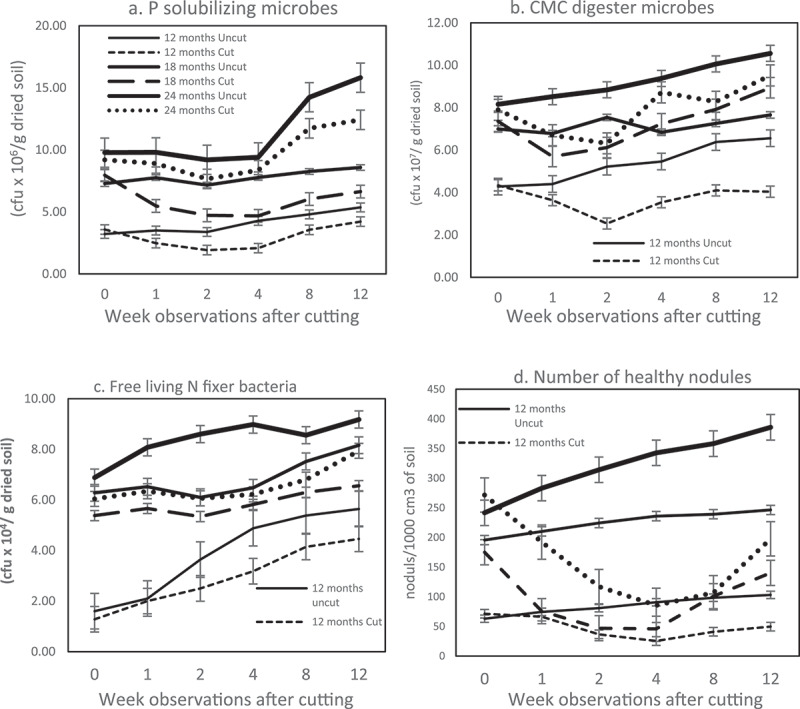
Microbes behavior in rhizosphere after cutting; (a) Phosphate solubilizer (PS), (b) Cellulose Microbes (CMC), (c) Biological Nitrogen Fixation – Free Living (BNF-FL), (d) Effective nodule represented by rhizobia. All functional groups population deplete a week after felling. The least affected is cellulose degrader (CMC) (Figure 3(a)) while the most impacted is rhizobia which are colonized in the root nodules (Figure 3(d)). (Solid line: uncut trees; dotted line: cut trees).

Figure 3. demonstrates that the population of all functional groups in the rhizosphere was drastically reduced due to cutting the host trees. The population reduction continued until the fourth week when the lowest population level was observed. Among the four groups of microbes analyzed, rhizobia (Figure 3(d)) were the least influenced by tree cutting.

### Age of cutting trees factor

Table 3 demonstrates the inconsistent influence of tree cutting age on soil microbial populations in the rhizosphere. The most impacted population was rhizobia, which decreased in the fourth week by 72–80%, whereas the most persistent groups were BNF-FL and CMC degraders; in the fourth week, their diminution was only 10–34% and 16–34%, respectively. (Table 3).

**Table 3.** Diminution of the soil-microbe population in the rhizosphere of stumps compared to that in uncut stands of a similar age (%)*

Week of sampling	PSM			CMC degraders			BNF-FL			Effective nodules		
	12	18	24	12	18	24	12	18	24	12	18	24
	Month			Month			Month			Month		
1	29.14	29.38	9.18	4.76	13.19	21.53	4.76	13.19	21.53	10.99	63.94	32.13
2	43.20	34.08	16.74	31.32	12.46	29.53	31.32	12.46	29.53	54.70	79.06	62.70
4	51.40	39.85	11.06	34.84	16.15	30.73	34.84	10.19	30.73	72.03	80.51	75.03
8	25.83	26.88	17.56	23.05	14.60	20.56	23.05	16.22	20.56	58.33	57.78	70.18
12	21.27	22.61	21.49	20.92	14.02	13.51	20.92	19.61	13.51	51.94	43.10	48.73

*percentage of diminution = [(uncut population – cut population)/uncut population] x 100%.

## Discussion

### Alteration of rhizosphere environment

Our study found that cutting of *C. calothyrsus* stands with resting stumps of 50–70 cm causes a significant change in the rhizosphere properties, such as reducing the TSS (Figure 1) and increasing soil acidity (Figure 2) in the root zone. This evidence proved a strong correlation between shoot and root systems, as the two play vital roles in plant life. Above ground, photosynthetic tissue captures carbon and provides the energy needs for all plant tissues, including the roots. The main products of photosynthesis are sugar compounds, some of which are the main contributors to root exudates secreted into the rhizosphere [3,13,17]. Hence, sugar is the most abundant organic compound in the biosphere [3]. Cutting removes all parts of the tree functioning in photosynthesis, consequently dramatically reducing the release of TSS to the rhizosphere (Table 2).

**Table 2.** Percentage of TSS depletion due to tree cutting

Week of observation	Plant’s age of cutting		
	12 month	18 month	24 month
0	15.04	1.84	1.23
1	52.98	31.27	40.64
2	63.89	63.13	62.43
4	80.87	70.95	69.51
8	63.61	49.56	60.95
12	39.61	41.65	57.29

Another impact of tree cutting on the rhizosphere observed in this study was the rapid change in soil acidity. The pH decreased drastically in the stump rhizosphere in the second week following tree cutting. The pH of the rhizosphere of *C. calothyrsus* decreased to < 5.0 from the second to the fourth week after cutting (Figure 2). However, the mechanisms and reasons for this decrease are still unclear. A previous study conducted by 28,showed a contradictory result; it was found that several factors could lead to increased soil pH following clear-cutting, one of which is the acceleration of organic material decomposition.

### The dynamics of soil microbial functional groups

In this study, all soil microbes (Figure 3) were sensitive to environmental changeability, primarily TSS reduction (Figure 1) and pH alteration (Figure 2). This is because TSS plays an important role as a C and energy source for the soil community. All culturable microbes (Figure 3) analyzed in this study were categorized as copiotrophs because they were depleted once the carbon source ceased due to tree cutting. Copiotroph microorganisms are strongly influenced by nutrients, especially C, in their environment [29]. This result conforms with earlier studies on clear-cutting techniques in forest harvesting, which have reported a significantly altered structure of soil bacterial communities as a result of depleted microbial biomass carbon, with a 17–33% reduction in varied forest soil [23]. Cutting directly changes the soil microclimate [30], litter quality and quantity, and decreases soil organic carbon and soil nutrient availability [31].

This study found that when the photosynthetic process was completely suspended by tree cutting, it disrupted the supply of nutrients and energy sources for soil microbial communities in the rhizosphere. This corroborates the findings of Henesmaa et al. (2005), who found that tree-felling diminished photosynthetic carbon flow from the tree into the soil. A study on the rhizosphere of stumps immediately after tree cutting showed sudden changes in the availability and composition of the C-affected-microbial communities in the rhizosphere. When the trees were felled, C flow from the roots to the soil stopped, influencing the root-associated microbial population [Henesmaa et al. 2005). 30,found that the direct impact of tree cutting on soil microbial physiology was due to the removal of organic matter. Cutting changes the carbon and nitrogen content and nutrient availability, leading to a low-resource environment.

In this study, the depletion of TSS due to cutting caused a dramatic decline in BNF-FL and rhizobia in the rhizosphere (Figures 3(c,d)]. The activities of NF microbes, either free-living or symbiotic, are strongly controlled by the concentration of carbon compounds in the rhizosphere [9]. Tree-cutting is thought to cut off the supply of root exudates to rhizobia, causing some roots to die. It can be concluded that since rhizobia are microbes living inside the inner tissue of the *C. calothyrsus* root system, they were directly affected by severe damage to the host plants.

The second most impacted microbes were CMC degraders inhabiting the rhizosphere of 12-month-old *C. calothyrsus*, with a reduction of up to 51% (Table 3), whereas PS microbes and BNF-FL were depleted by 11 to 39% (Table 3). The other groups (CMC, PS, and BNF-FL) were presumed to rely on their carbon source for root exudates and the SOM decomposition process as an alternative to the C supply. Soil organic matter is assumed to be supplied from dead roots and decomposed tree-felling matter. However, as a limitation of this study, there was no observation of SOM content after tree cutting; hence, the exact C source for CMC degrader, BNF-FL, and PS microbes cannot be justified.

In this study, soil microbes were found to be sensitive to soil pH alteration. Rhizosphere-pH depletion of approximately 1 to 1.5 units (Figure 2) after cutting caused a population decline of all soil microbes colonizing the rhizosphere (Figure 3). This evidence followed studies by 32,and 33, where the composition of soil bacterial communities was intensely defined by soil pH [34]. The interval varies by species, and the range for various bacteria is 1.5 to 3 pH units [34]. The apparent direct influence of pH on bacterial community composition is probably due to the narrow pH range required for the optimal growth of bacteria [35]. A slight change in pH affects more than half of the bacterial activities [32].

36,stated that pH alteration strongly influences the microbial population in the soil. Changes in pH can either accelerate or inhibit growth and in extreme conditions, cause eradication. Soil pH affects the structure and function of the soil microbial community both directly and indirectly. The direct interaction of hydrogen ions (H^+^) with microbial cells may influence microbial communities in several ways, including the disruption of cell membranes, altered enzyme production, and limited reproduction. This equates to a reduced overall microbial function in the health and productivity of soils [37]. [38] explained that pH is a critical environmental parameter for microbial growth because the stability of the lipid membrane and proteins depend strongly on pH. Indirectly, changes in soil pH affect the bacterial efficiency to catabolize cysteine, aspartic acid, lysine, and arginine [39]. Increasing acidity also changes the availability of some elements (Cu, Al, Fe) that may be toxic to soil microbial communities [40].

Similar to the TSS reduction, symbiotic NFs seemed to accept the most severe impact of pH depletion (Figure 3(d)). Four weeks after cutting, *C. calothyrsus* stumps lost 72–80% of effective nodules from the rhizosphere (Table 3) due to pH depletion of approximately 1–1.5 units. 41,found that rhizobia flourish and function best in neutral to basic soil pH. At a natural soil pH level, 6–7 legume roots naturally develop an association with rhizobia in the soil and symbiotically fix N. In this study, cutting of *C. calothyrsus* caused pH depletion to a unit level of 4.9 in the second week. Low soil pH can significantly decrease nodule number, nodule function, and N-fixing capabilities within legume roots [42]. A study on soybeans planted in soil with a pH level < 5 and > 6 reported that the plants cultivated at pH < 5 lost approximately 40–60% of the nodule number, compared to that of the plants in pH > 6 [43]. Low pH can inhibit nodulation by limiting the ability of legumes to secrete the required signals involved in nodule formation in the rhizosphere [44,45].

38 hypothesized that microorganisms develop unique adaptations to detrimental situations. For example, resource-depleted microbes change the habitat environment to reduce their population. This phenomenon is known as ecological suicide. A study on the bacteria *Paenibacillus* sp. reported modifying the environmental pH to such a degree that it could rapidly kill the whole population. In this study, it is still unclear whether the increase in acidity to diminish the microbial population was controlled by plants or by the microbes themselves.

All of the observed functional groups showed an increased population in the eighth week (Figure 3) when the soil pH reached a level of 6 (Figure 2). The concentration of TSS began to improve by the eighth week and continued to increase until the twelfth week of observation (Figure 1). The growth of new shoots, which serve as the plant’s photosynthetic area, is thought to be the cause of this improvement.

### Survival strategy for regrowing

*C. calothyrsus* survive to develop new shoots within several weeks after cutting. It is assumed that survival mechanisms are controlled in the rhizosphere. This study found that there are two mechanisms that reduce sugar deposits by limiting the release of TSS (Figure 1) and increasing soil acidity (Figure 2) to manage the magnitude, structure, and composition of rhizobia, BNF-FL, CMC degraders, and PS microbe populations in the rhizosphere (Figure 3). According to 46, a stump must save its root exudates by recapturing substances from the rhizosphere after being cut. The depletion of TSS after tree cutting in this study (Figure 1) is presumably a stump-survival strategy. However, to determine a clear mechanism for decreasing TSS in this study, further studies are required.

Plants select the rhizosphere community by attracting beneficial or removing detrimental microbes [2]. Interactions between plants and microbes change from partner to competitor, corresponding to the plants’ growth and environmental circumstances [47]. The depletion of soil pH immediately a week after cutting is assumed to be a strategy of *C. calothyrsus* in assembling the harmless composition and structure of their rhizosphere communities. According to 48, plants increase the acidity of the rhizosphere to eliminate competitors from their root zones. Roots release protons to the rhizosphere, which depresses rhizosphere pH [48].

## Conclusion

This study proves that cutting significantly reduces the flux of sugars below ground and causes rapid acidification of the soil. Total soil sugar depletion is presumed to be a mechanism by which *C. calothyrsus* survives and regrows after cutting by saving the photosynthetic products. However, depletion disrupts the supply of nutrients and energy sources for soil microbial communities in the rhizosphere. This results in significant shifts in the size and structure of the rhizosphere microbial community. Increasing soil acidity is another survival strategy to limit close competitor populations in the rhizosphere. This was proven by the fact that rhizobia living inside the inner tissue of the *C. calothyrsus* root were the most significantly diminished. Rhizosphere susceptibility to tree-cutting disturbances did not correlate with tree age. The rhizosphere started to recover in the 8^th^ week of observation. The study confirms that *C. calothyrsus* is a proper species for development in the CHS energy estate.
